# Long non-coding RNA MSC-AS1 facilitates the proliferation and glycolysis of gastric cancer cells by regulating PFKFB3 expression

**DOI:** 10.7150/ijms.51947

**Published:** 2021-01-01

**Authors:** Xianzhen Jin, Lina Qiao, Hui Fan, Chunyan Liao, Jianbao Zheng, Wei Wang, Xiuqin Ma, Min Yang, Xuejun Sun, Wei Zhao

**Affiliations:** 1 Department of General Surgery, The First Affiliated Hospital of Xi'an Jiaotong University, Xi'an 710061, P.R. China.; 2 Department of Nursing, Hanzhong Central Hospital, Hanzhong 723000, P.R. China.; 3 Department of Nursing, Xianyang Hospital, Yan'an University, Xianyang 712000, P.R. China.

**Keywords:** Gastric cancer, LncRNA, MSC-AS1, PFKFB3, Proliferation, Glycolysis

## Abstract

Long non-coding RNA musculin antisense RNA 1 (lncRNA MSC-AS1) has been recognized as an oncogene in pancreatic cancer, hepatocellular carcinoma, nasopharyngeal carcinoma, and renal cell carcinoma. However, the functional significance of MSC-AS1 and its underlying mechanism in gastric cancer (GC) progression remain unclear. In this study, we demonstrated that the expression of MSC-AS1 in GC tissues was significantly higher than that in non-tumor tissues. Moreover, the elevated level of MSC-AS1 was detected in GC cells (MKN-45, AGS, SGC-7901, and MGC-803) compared to normal GES-1 gastric mucosal cells. The cancer genome atlas (TCGA) data further indicated that the high level of MSC-AS1 was closely correlated with advanced tumor stage and poor prognosis of GC. Next, we revealed that MSC-AS1 knockdown inhibited the proliferation, glucose consumption, lactate production, and pyruvate production of MGC-803 cells. Conversely, MSC-AS1 overexpression enhanced the proliferation and glycolysis of AGC cells. Mechanistically, modulating MSC-AS1 level affected the expression of 6-phosphofructo-2-kinase/fructose-2,6-biphosphatase 3 (PFKFB3), but did not impact the levels of hexokinase 2 (HK2) and pyruvate kinase M2 (PKM2) in GC cells. Based on this, we reversed the MSC-AS1 knockdown-induced the inhibition of cell proliferation and glycolysis by restoring PFKFB3 expression in MGC-803 cells. In conclusion, MSC-AS1 facilitated the proliferation and glycolysis of GC cells by maintaining PFKFB3 expression.

## Introduction

Gastric cancer (GC), one of the most common malignancies, has a high morbidity and mortality worldwide 1. Currently, surgical resection remains the leading therapeutic strategy for GC 2. Chemotherapy, targeted therapy, and immunotherapy are preferred options for advanced GC 2. But, the clinical outcomes of GC patients have not shown satisfactory improvement 3, 4. Thus, it is essential to investigate the underlying mechanisms involved in GC's initiation and progression and explore novel therapeutic targets for GC.

Previous studies have demonstrated that the human genome encodes long non-coding RNAs (lncRNAs; ≥ 200 nucleotides in length), which have a limited or no ability to encode proteins 5. Currently, increasing evidence supports that lncRNAs act as vital regulators in cellular development, differentiation, and various other biological processes 6. Importantly, lncRNAs perform critical functions in the initiation and progression of human cancers 7-14. For instance, lncRNA PVT1 is highly expressed in GC and prominently correlated with patients' poor clinical outcomes 15. PVT1 directly binds to signal transducer and activator of transcription 3 (STAT3) and maintains its protein stability in GC cells 15. The overexpressed lncRNA HOXC-AS3 promotes GC cells' proliferation and migration by interacting with Y-box binding protein 1 (YBX1) 16. Besides, lncRNA MACC1-AS1 enhances GC cells' proliferation and glycolysis via the AMPK/Lin28 pathway-mediated MACC1 upregulation 17. LncRNA musculin antisense RNA 1 (MSC-AS1) has been recognized as an oncogene in pancreatic ductal adenocarcinoma (PDAC) 18, hepatocellular carcinoma (HCC) 19, nasopharyngeal carcinoma (NPC) 20, and renal cell carcinoma (RCC) 21. MSC-AS1 functions as a molecular sponge for miR-29b-3p to increase cyclin-dependent kinase 14 (CDK14) expression, which facilitates PDAC cell proliferation 18. MSC-AS1 contributes to cell proliferation and invasion in RCC via activating the miR-3924-mediated Wnt/β-catenin signaling pathway 21. MSC-AS1 is reported to promote the proliferation, apoptosis resistance, invasion, and epithelial-to-mesenchymal transition (EMT) of NPC cells by regulating the miR-524-5p/nuclear receptor subfamily 4 group A member 2 (NR4A2) axis 20. Moreover, MSC-AS1 accelerates HCC progression through enhancing the expression of phosphoglycerate kinase 1 19. However, it is unclear whether and how MSC-AS1 participates in GC progression.

In the present study, we determined the expression difference of MSC-AS1 between GC and noncancerous tissues. The clinical significance of MSC-AS1 in GC was analyzed according to the cancer genome atlas (TCGA) data. Then, we explored the effects of MSC-AS1 on GC cell proliferation and glycolysis and investigated its possible underlying mechanism. Our data suggested that MSC-AS1 was highly expressed in GC and facilitated cell proliferation and glycolysis by enhancing 6-phosphofructo-2-kinase/fructose-2,6-biphosphatase 3 (PFKFB3) expression.

## Materials and methods

### Human tissue samples

Eighty GC tissue samples and 80 tumor-adjacent tissue samples were obtained from patients who signed informed consent forms and received surgical resection at 1^st^ Affiliated Hospital of Xi'an Jiaotong University. All specimens were pathologically confirmed as GC and stored at -80°C for subsequent analysis. This study was approved by the Ethics Committee of the First Affiliated Hospital of Xi'an Jiaotong University. The clinical features of the patients were described in our previous study 22.

### Cell culture and transfection

The human GC cells (MKN-45, AGS, SGC-7901, and MGC-803) and normal GES-1 gastric mucosal cells were maintained in our lab under standard culture conditions 22. MSC-AS1 shRNA (shMSC-AS1-1 and shMSC-AS1-2) and non-targeting RNA (NT shRNA) were obtained from GenePharma (Shanghai, China). The pcDNA3.1-PFKFB3 and pcDNA3.1-MSC-AS1 vectors were constructed by inserting the cDNA products of PFKFB3 and MSC-AS1 into the expression vector pcDNA3.1 (Invitrogen, Carlsbad, CA, USA). The plasmids were delivered into GC cells using Lipofectamine 2000 (Invitrogen) according to the manufacturer's instructions.

### Real-time quantitative PCR (qRT-PCR)

Total RNAs were isolated from tissues and cells with TRIzol reagent (Invitrogen), and reverse-transcribed into cDNA using the TIANScript RT Kit (Tiangen Biotech, Beijing, China). qRT-PCR was performed using SYBR Green PCR Master Mix (Takara, Dalian, China) in the CFX96 Touch™ Real-Time PCR Detection System (Bio-Rad Laboratories, Hercules, CA, USA). The expression level of mRNA was normalized to GAPDH expression using the 2^-ΔΔCt^ method. The sequences of the primers were listed as follows. MSC-AS1 forward 5′-AAGCAACAACTGTCTGGCCT-3′, reverse 5′-TGATGCCAGCAAATTGGTGC-3′; 6-phosphofructo-2-kinase/fructose-2,6-biphosphatase 3 (PFKFB3) forward 5′- CAGTTGTGGCCTCCAATATC-3′, reverse 5′- GGCTTCATAGCAACTGATCC-3′; hexokinase 2 (HK2) forward 5′- ATTGTCCAGTGCATCGCGGA-3′, reverse 5′- AGGTCAAACTCCTCTCGCCG-3′; pyruvate kinase M2 (PKM2) forward 5′-CAGAGGCTGCCATCTACCAC-3′, reverse 5′-CCAGACTTGGTGAGGACGAT-3′; GAPDH forward 5′-AGCAAGAGCACAAGAGGAAG-3′, reverse 5′-GGTTGAGCACAGGGTACTTT-3′.

### Cell proliferation analysis

The proliferation ability of transfected cells was analyzed using the Cell Counting Kit-8 (CCK-8; Dojindo Laboratories, Dojindo, Japan) assay and 5-ethynyl-2′-deoxyuridine (EdU) staining. For CCK-8 assay, the transfected cells (2×10^3^ cells per well) were seeded into 96-well plates. 10 μL of CCK-8 reagent was added into the plates and incubated for an additional 2 h at 37 °C. Optical-density values at 450 nm were measured with a microplate reader (Thermo Fisher Scientific, Waltham, MA, USA). The Cell-Light™ EdU Apollo®488 *In vitro* Imaging Kit (RIBOBIO, Guangzhou, China) was used for EdU staining, as previously described 23.

### Detection of Glucose consumption, lactate production, and pyruvate production

The transfected cells (1×10^6^ cells per well) were seeded into 6-well plates and incubated for 24 h at 37 °C under indicated treatment. The culture medium was collected and subjected to a glucose assay kit (ab136955, Abcam, Cambridge, MA, USA), lactate assay kit (ab65331, Abcam), and pyruvate assay kit (ab65342, Abcam) for measuring glucose consumption, lactate production, and pyruvate production according to the manufacturer's instructions.

### Western blotting

Cells were lysed with the RIPA lysis buffer (Beyotime, Shanghai, China) containing a proteasome inhibitor (PI, Roche, Indianapolis, IN, USA) and a phosphatase inhibitor (Thermo Fisher Scientific, Waltham, MA, USA). 20 μg of each protein were resolved by SDS-PAGE and transferred to PVDF membranes (Millipore, Bedford, MA, USA). The membranes were blocked with 5% skimmed milk for 1 h at room temperature and then incubated with the PFKFB3 antibody (ab181861, Cambridge, MA, USA) and GAPDH antibody (ab8226, Abcam) at 4 °C overnight. The next day, we incubated the membranes with suitable horseradish peroxidase (HRP)-conjugated secondary antibody (Beyotime) for 1 h at room temperature. The blots were then visualized with the ECL reagent (Millipore) and detected by an Amersham Imager 600 (GE Healthcare Life Sciences, Pittsburgh, PA, USA). The ImageJ software (1.46; National Institutes of Health, Bethesda, MD, USA) was used for semi-quantitative immunoblots analysis.

### Statistical analysis

The data of at least three independent experiments were presented as the mean ± SD and analyzed with GraphPad Prism 8.0 (GraphPad Inc., San Diego, CA, USA). The one-way ANOVA and Student's *t*-test were performed to calculate the differences among the groups. *P*<0.05 was considered significant.

## Results

### The elevated level MSC-AS1 associated with the poor prognosis of GC

**Figure 1:**
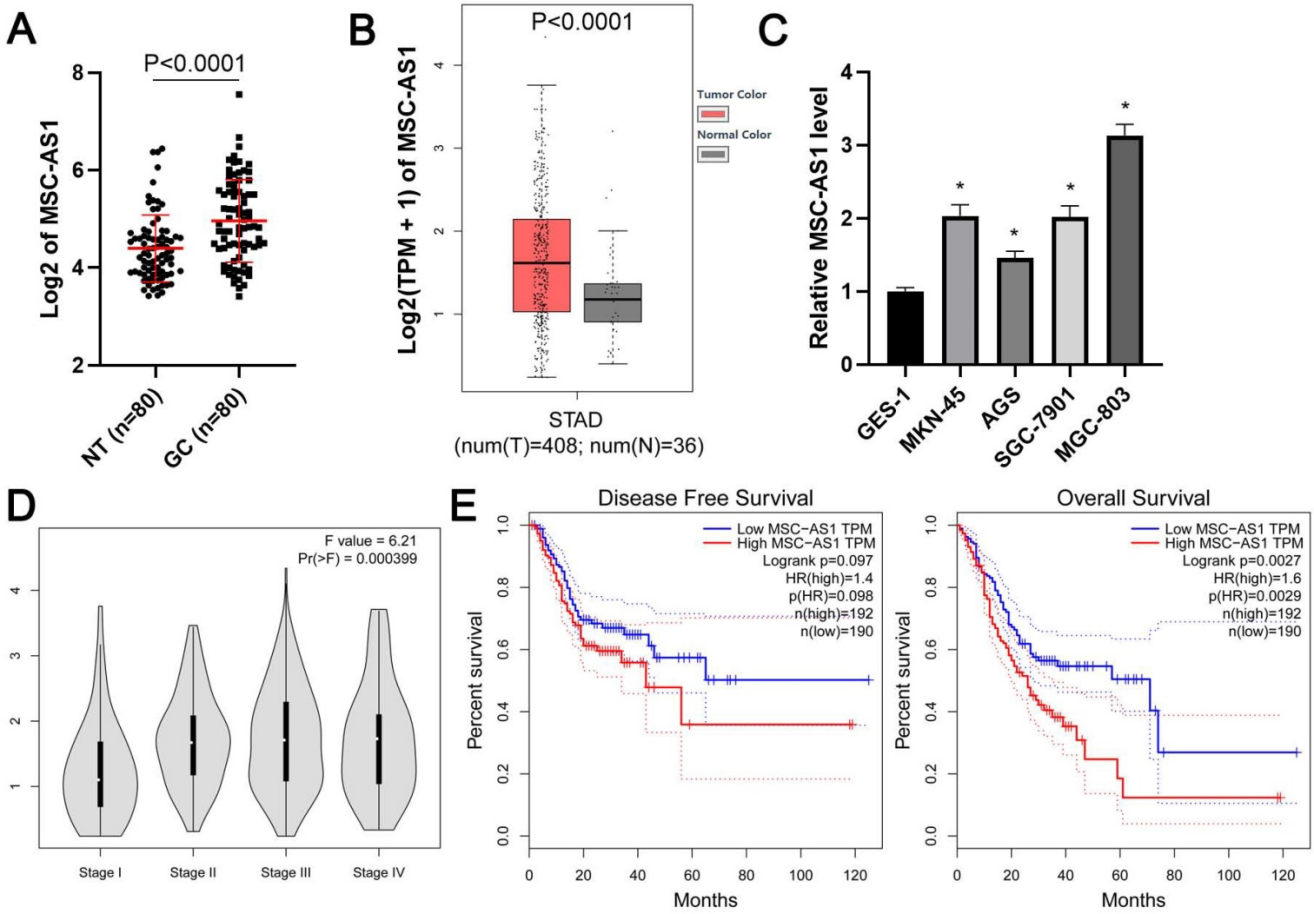
** The expression and prognostic significance of MSC-AS1 in GC.** (A) The expression of MSC-AS1 in 80 pairs of GCs and adjacent nontumor (NT) tissues. (B) TCGA data from GEPIA platform revealed an upregulated expression of MSC-AS1 in GC tissues. (C) The levels of MSC-AS1 in GC cells (MKN-45, AGS, SGC-7901, and MGC-803) were compared with normal GES-1 gastric mucosal cells. (D) TCGA data from the GEPIA platform indicated that the high level of MSC-AS1 correlated with GC's advanced tumor stage. (E) TCGA data from the GEPIA platform suggested that GC patients with an increased expression of MSC-AS1 in tumor tissues had an apparent lower overall survival and disease-free survival. *P<0.05.

First, qRT-PCR was carried out to determine the expression difference of MSC-AS1 between GC and adjacent nontumor tissues. Our results showed that the level of MSC-AS1 in GC tissue samples was significantly higher than that in the tumor-adjacent tissue samples (*P*<0.0001, Figure 1A). Moreover, TCGA data from the gene expression profiling interactive analysis (GEPIA) platform 24 and gene expression omnibus (GEO) datasets (GSE54129, GSE65801, and GSE13911) from lnCAR platform 25 consistently revealed the upregulated expression of MSC-AS1 in GC tissues compared to normal tissues (*P*<0.05, Figure 1B and Supplementary Figure 1). MSC-AS1 expressed at higher levels in GC cells (MKN-45, AGS, SGC-7901, and MGC-803) as compared with normal GES-1 gastric mucosal cells (*P*<0.05, Figure 1C). Notably, the analysis of TCGA data using GEPIA platform indicated that a high MSC-AS1 level in GC tissues was correlated with advanced tumor stage and predicted poor prognosis for the patients (*P*<0.05, Figure 1D and 1E).

### MSC-AS1 affected the proliferation and glycolysis of GC cells

**Figure 2:**
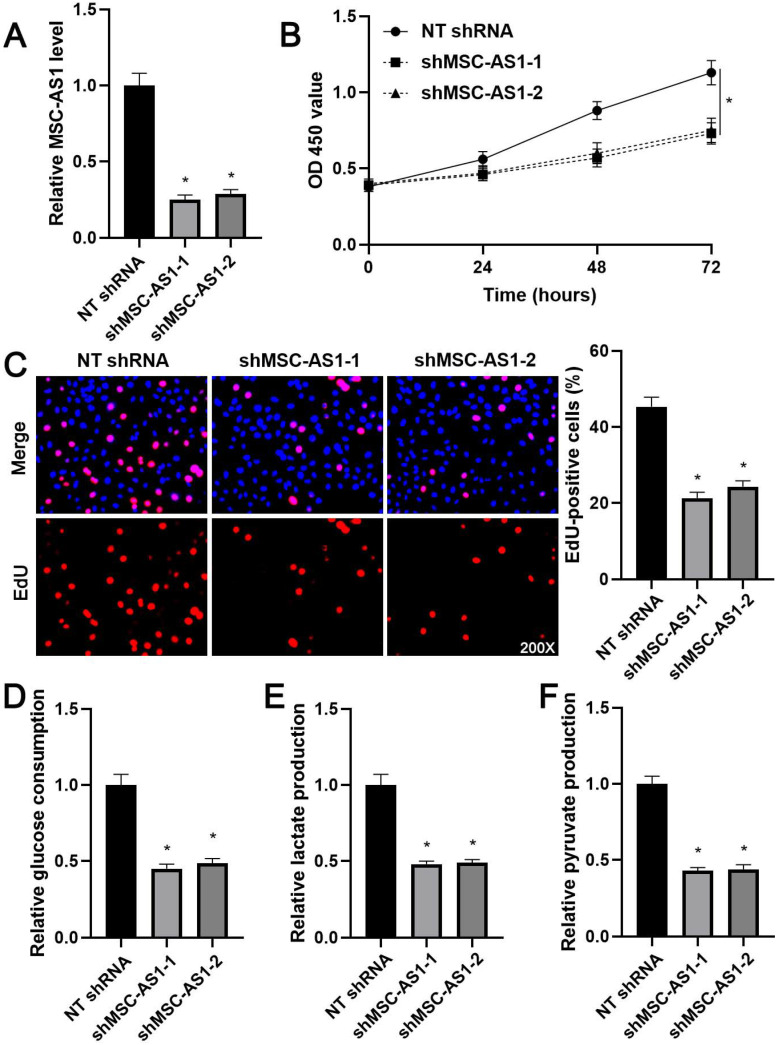
** MSC-AS1 knockdown inhibits the proliferation and glycolysis of MGC-803 cells.** (A) MGC-803 cells were transfected with non-targeting (NT) shRNA or MSC-AS1 shRNAs (shMSC-AS1-1 and shMSC-AS1-2) and assessed by qRT-PCR for MSC-AS1 expression. (B) A CCK-8 assay indicated that MSC-AS1 knockdown inhibited the viability of MGC-803 cells. (C) An EdU staining revealed that MSC-AS1 silencing repressed the proliferation of MGC-803 cells. (D-F) The glucose consumption, lactate production, and pyruvate production were decreased after MSC-AS1 knockdown in MGC-803 cells. *P<0.05.

**Figure 3:**
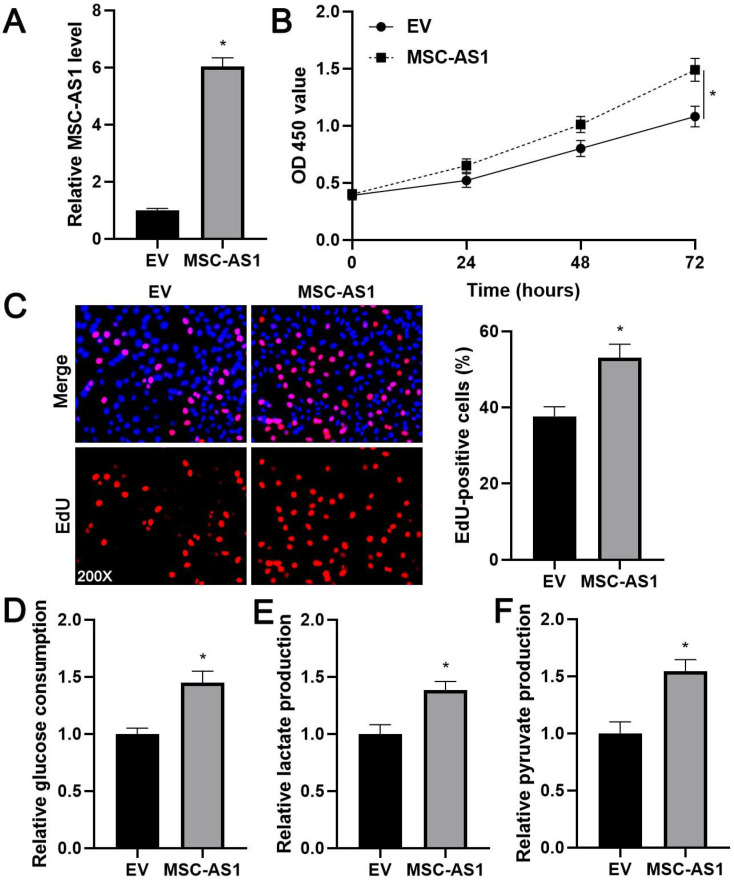
** MSC-AS1 overexpression promotes the proliferation and glycolysis of AGS cells.** (A) AGS cells were transfected with empty vector (EV) or pcDNA3.1-MSC-AS1 and assessed by qRT-PCR for MSC-AS1 expression. (B) A CCK-8 assay indicated that MSC-AS1 overexpression promoted the viability of AGS cells. (C) An EdU staining revealed that MSC-AS1 silencing facilitated the proliferation of AGS cells. (D-F) The glucose consumption, lactate production, and pyruvate production were enhanced after MSC-AS1 overexpression in AGS cells. *P<0.05.

Next, we investigated the proliferation and glycolysis of GC cells after modulating the MSC-AS1 level. The expression of MSC-AS1 was significantly knocked down in MGC-803 cells, which expressed the relative highest level of MSC-AS1, using two independent shRNAs (*P*<0.05, Figure 2A). The CCK-8 assay revealed that MGC-803 cells' viability was markedly suppressed by MSC-AS1 knockdown (*P*<0.05, Figure 2B). The proliferation of MGC-803 cells was prominently decreased by MSC-AS1 silencing, as suggested by EdU staining (*P*<0.05, Figure 2C). Furthermore, the depletion of MSC-AS1 remarkably reduced glucose consumption, lactate production, and pyruvate production in MGC-803 cells (*P*<0.05, Figure 2D-2F). Then, MSC-AS1 was overexpressed in AGS cells, which expressed the relative lowest level of MSC-AS1, using the expression plasmid (*P*<0.05, Figure 3A). Conversely, MSC-AS1 overexpression significantly enhanced the proliferation and glycolysis of AGS cells (*P*<0.05, Figure 3B-3F).

### MSC-AS1 regulated PFKFB3 expression in GC cells

**Figure 4:**
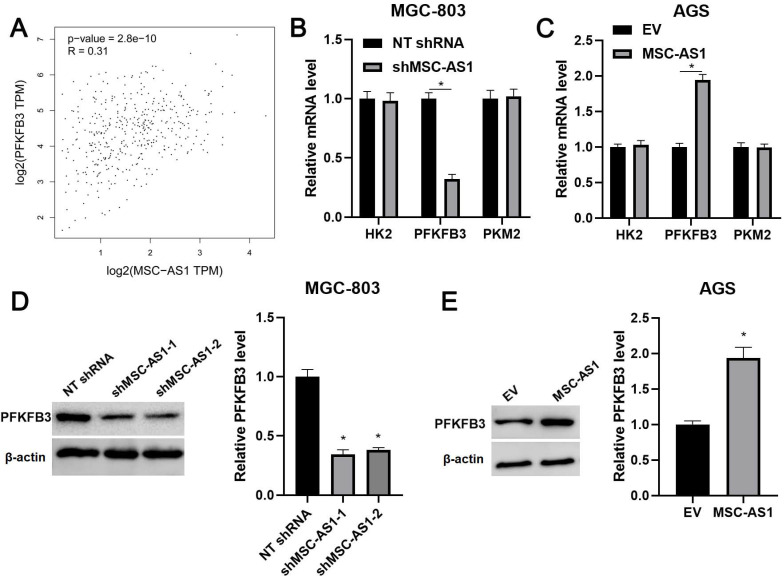
** MSC-AS1 regulates PFKFB3 expression in GC cells.** (A) TCGA data from the GEPIA platform indicated a positive correlation between MSC-AS1 and PFKFB3 mRNA expression in GC tissues. (B) MGC-803 cells were transfected with non-targeting (NT) shRNA or MSC-AS1 shRNAs (shMSC-AS1-1 and shMSC-AS1-2) and assessed by qRT-PCR for HK2, PFKFB3, and PKM2 mRNA expression. (C) AGS cells that were transfected with empty vector (EV) or pcDNA3.1-MSC-AS1 and assessed by qRT-PCR for HK2, PFKFB3, and PKM2 mRNA expression. (D) MSC-AS1 knockdown reduced the level of PFKFB3 protein in MGC-803 cells. (E) MSC-AS1 overexpression increased the expression of PFKFB3 protein in AGS cells. *P<0.05.

KEGG pathway analysis of GEO) datasets (GSE54129, GSE65801, and GSE13911) from lnCAR platform suggested the involvement of MSC-AS1 in GC's metabolic pathways (Supplementary Figure 2). Since glycolysis is regulated by three key rate-limiting enzymes, including HK2, PFKFB3, and PKM2 26. Thus, the correlations between MSC-AS1 and HK2 mRNA, PFKFB3 mRNA, and PKM2 mRNA were determined in GC tissues from TCGA database using GEPIA platform. Our data found that MSC-AS1 was positively correlated with PFKFB3 mRNA in GC tissues (*P*<0.0001, Figure 4A), but did not associate with HK2 mRNA and PKM2 mRNA (Supplementary Figure 3). Then, the levels of HK2 mRNA, PFKFB3 mRNA, and PKM2 mRNA were detected in GC cells after modulating MSC-AS1 expression. As shown in Figure 4B and 4C, MSC-AS1 knockdown decreased the PFKFB3 mRNA level in MGC-803 cells, while MSC-AS1 overexpression increased the expression of PFKFB3 mRNA in AGS cells (*P*<0.05). However, MSC-AS1 did not impact HK2 mRNA and PKM2 mRNA levels in GC cells (Figure 4B and 4C). Consistently, MSC-AS1 positively regulated PFKFB3 protein expression in GC cells as suggested by western blotting analysis (*P*<0.05, Figure 4D and 4E). TCGA database analysis using GEPIA platform indicated that PFKFB3 mRNA expression in GC tissues was significantly higher than that in normal tissues (*P*<0.0001, Supplementary Figure 4A). Moreover, PFKFB3 mRNA expressed at higher levels in GC cells (MKN-45, AGS, SGC-7901, and MGC-803) as compared with GES-1 cells (*P*<0.05, Supplementary Figure 4B).

### PFKFB3 mediated the role of MSC-AS1 in GC cells

**Figure 5:**
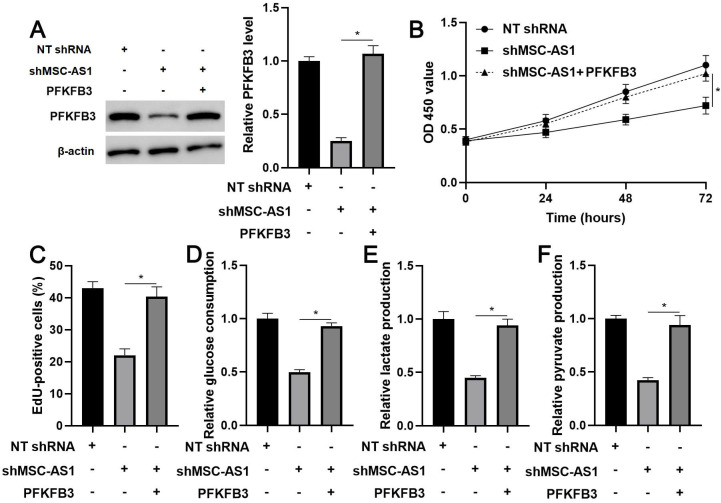
** PFKFB3 restoration reverses the effects of MSC-AS1 knockdown on MGC-803 cells.** (A) MSC-AS1 knockdown MGC-803 cells were transfected with pcDNA3.1-PFKFB3 to restore PFKFB3 expression. (B) CCK-8, (C) EdU, (D) glucose consumption, (E) lactate production, and (F) pyruvate production assays were performed to detect the proliferation and glycolysis of MGC-803 cells. *P<0.05.

The expression of PFKFB3 was restored in MSC-AS1 knockdown MGC-803 cells (*P*<0.05, Figure 5A) to confirm whether PFKFB3 was a downstream effector of MSC-AS1. A CCK-8 analysis indicated that the viability of MSC-AS1 silenced MGC-803 cells was significantly promoted by PFKFB3 restoration (*P*<0.05, Figure 5B). Besides, PFKFB3 restoration markedly reversed MSC-AS1 knockdown-induced the proliferation inhibition in MGC-803 cells (*P*<0.05, Figure 5C). Significantly, MSC-AS1 silencing-reduced glucose consumption, lactate production, and pyruvate production were prominently enhanced by the re-expression of PFKFB3 in MGC-803 cells (*P*<0.05, Figure 5D-5F).

## Discussion

The aberrant level of lncRNAs has been detected in GC tissues and serum samples, which may be used as potential diagnostic and prognostic biomarkers 27. For example, the high expression levels of lncRNA HOXC-AS3 are correlated with lower overall survival in GC patients, as demonstrated by Kaplan-Meier survival analysis 16. Compared to carcinoembryonic antigen, the exosomal lncUEGC1 in the serum shows a higher diagnostic accuracy for discriminating GC patients from healthy individuals 28. The expression of MSC-AS1 is frequently overexpressed in PDAC, HCC, RCC, and NPC 18-21. Moreover, the high level of MSC-AS1 predicts poor clinical outcomes of patients with PDAC, HCC, and RCC 18, 19, 21. Here, we revealed an upregulated expression of MSC-AS1 in GC tissues and cells. TCGA data and GEO data further supported the highly expressed MSC-AS1in GC. Interestingly, TCGA data demonstrated that the elevated expression of MSC-AS1 was correlated with advanced tumor stage and poor prognosis of GC. Therefore, our results suggested MSC-AS1 as a promising prognostic marker in GC.

Previous studies have explored the biological function of MSC-AS1 in human cancers, including PDAC, HCC, RCC, and NPC 18-21. MSC-AS1 knockdown induces proliferation inhibition, apoptosis, and G1 phase arrest, and represses cell migration/invasion in HCC cells 19. MSC-AS1 participates in tumor progression via promoting cell proliferation and invasion in NPC and RCC 18, 20. Also, MSC-AS1 silencing significantly suppressed the proliferation of PDAC cells 18. In this study, loss-of and gain-of-function experiments indicated that MSC-AS1 positively regulated cell proliferation, glucose consumption, lactate production, and pyruvate production in GC cells. Thus, MSC-AS1 acted as an oncogene in GC.

Glycolysis, also known as the Warburg effect, is an intracellular metabolic process consisting of 10 consecutive enzymatic reactions that degrade one molecule of glucose into two pyruvates 29. Enhance glycolysis supports cancer cells' rapid growth, provides essential precursors for cell biosynthesis, and promotes tumor angiogenesis 26. HK2, PFKFB3, and PKM2 are identified initially as key glycolysis enzymes that participate in glucose metabolism 26, 30. A recent study reports that lncRNA AGPG enhances glycolysis activity and promotes tumor growth via maintaining PFKFB3 protein stability in esophageal squamous cell carcinoma (ESCC). Here, our results suggested that MSC-AS1 regulated PFKFB3 expression at the mRNA level. We found that MSC-AS1 enhanced the expression of PFKFB3 mRNA and protein in GC cells, but did not impact the levels of HK2 and PKM2. TCGA data also revealed the positive correlation between MSC-AS1 and PFKFB3 mRNA expression in GC tissues. Prior studies have demonstrated that PFKFB3 is highly expressed in GC and facilitated the proliferation, migration, and invasion of cancer cells 31, 32. After that, the role of PFKFB3 in the metabolic control of cancer cell glycolysis has been widely reported 33-35. Hypoxia-induced PFKFB3 and PFKFB4 play a vital role in the Warburg effect in GC cells 36. Our study further found that PFKFB3 restoration significantly enhanced the knockdown of MSC-AS1-attenuated GC cells' proliferation and glycolysis.

Collectively, our data explored the overexpression of MSC-AS1 in GC. Moreover, we demonstrated the significant roles of MSC-AS1 in GC cell proliferation and glycolysis. PFKFB3, which was positively regulated by MSC-AS1, mediated the role of MSC-AS1 in GC cells. Future work is needed to uncover the underlying mechanism involved in regulating PFKFB3 by MSC-AS1 in GC.

## Supplementary Material

Supplementary figures.

## Abbreviations

GC: gastric cancer
LncRNA: long non-coding RNA
STAT3: signal transducer and activator of transcription 3
YBX1: Y-box binding protein 1
MSC-AS1: musculin antisense RNA 1
PDAC: pancreatic ductal adenocarcinoma
HCC: hepatocellular carcinoma
NPC: nasopharyngeal carcinoma
RCC: renal cell carcinoma
CDK14: cyclin dependent kinase 14
EMT: epithelial-to-mesenchymal transition
NR4A2: nuclear receptor subfamily 4 group A member 2
PFKFB3: 6-phosphofructo-2-kinase/fructose-2,6-biphosphatase 3
HK2: hexokinase 2
PKM2: pyruvate kinase M2
ESCC: esophageal squamous cell carcinoma
