# Supplementary material for: Long non-coding RNA MSC-AS1 facilitates the proliferation and glycolysis of gastric cancer cells by regulating PFKFB3 expression
Source: Int J Med Sci. 2021 Jan 1;18(2):546–54. doi: 10.7150/ijms.51947 (PMC7757144; doi:10.7150/ijms.51947)
Supplement: Supplementary file 1 — Supplementary figures. [file ijmsv18p0546s1.pdf]

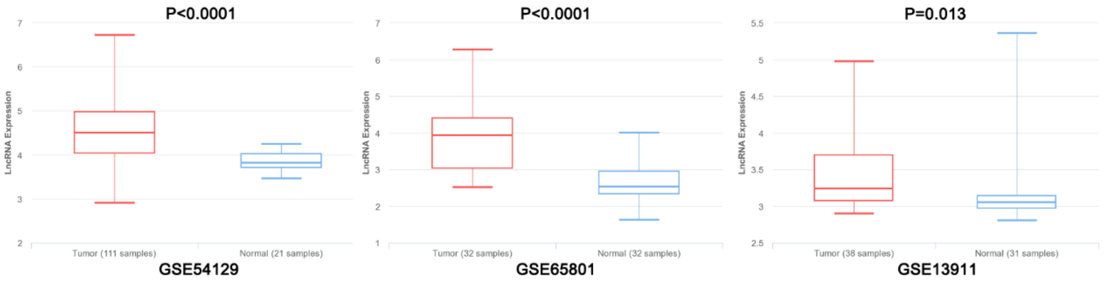

Supplementary Figure 1 The expression of MSC-AS1 in GC was analyzed with GEO datasets from lnCAR platform.

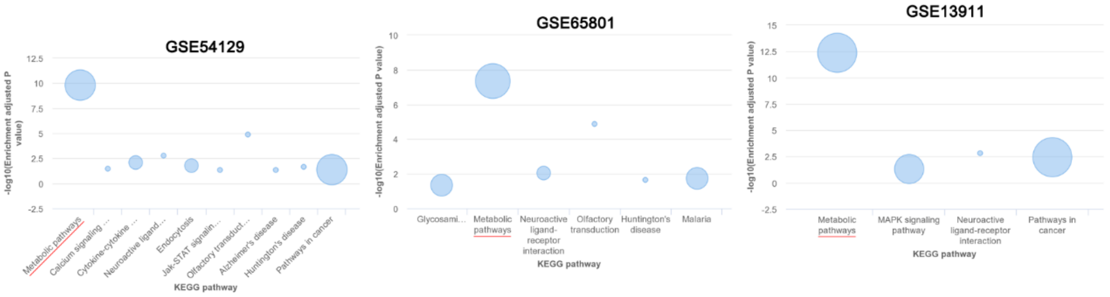

Supplementary Figure 2 KEGG pathway analysis of MSC-AS1 in GC was analyzed with GEO datasets from lnCAR platform.

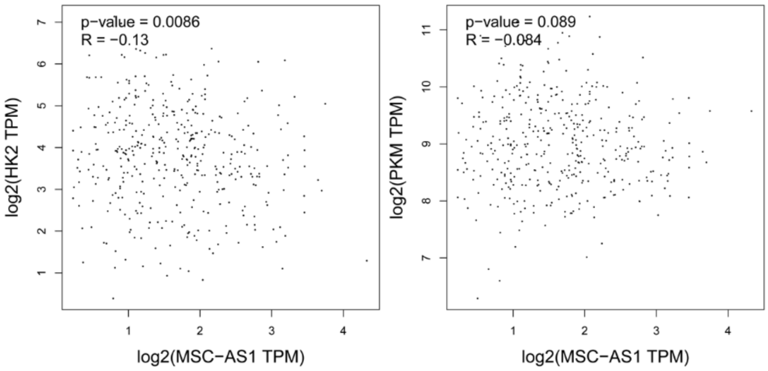

Supplementary Figure 3 The correlations between MSC-AS1 and HK2 mRNA and PKM2 mRNA expression in GC tissues from TCGA data.

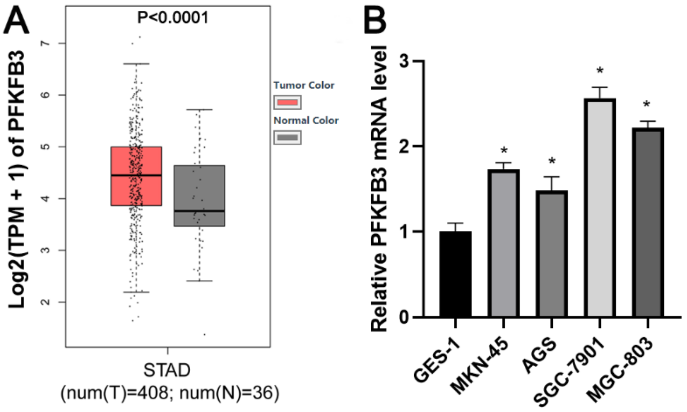

Supplementary Figure 4 The expression of PFKFB3 mRNA in GC. (A) TCGA data from the GEPIA platform revealed an upregulated expression of PFKFB3 mRNA in GC tissues. (B) The levels of PFKFB3 mRNA in GC cells (MKN-45, AGS, SGC-7901, and MGC-803) were compared with normal GES-1 gastric mucosal cells. *P<0.05.
